# The Influence of Improved Access to Alcohol-Based Hand Rub and Hand Hygiene Training in Healthcare Facilities on Hand Hygiene Adherence in Belize During COVID-19: June 2021–August 2022

**DOI:** 10.3390/ijerph22040514

**Published:** 2025-03-28

**Authors:** Kelsey McDavid, Anh N. Ly, Nicholas Bivens, Francis Morey, Gerhaldine Morazan, Russell Manzanero, Melissa Musa-Diaz, Alexandra Medley, Kristy O. Murray, Matthew J. Lozier

**Affiliations:** 1 Division of Foodborne, Waterborne, and Environmental Diseases, Centers for Disease Control and Prevention, 1600 Clifton Rd, Atlanta, GA 30333, USA; ngl7@cdc.gov (K.M.); wfu2@cdc.gov (M.J.L.); 2 Department of Pediatrics, National School of Tropical Medicine, Baylor College of Medicine and Texas Children’s Hospital, 1102 Bates Ave, Houston, TX 77030, USA; 3 Belize Ministry of Health and Wellness, East Block Building, National Assembly, Bliss Parade, Belmopan, Belize; 4 Department of Pediatrics, Emory University and Children’s Healthcare of Atlanta, 2015 Uppergate Dr., Atlanta, GA 30329, USA; 5 United States Public Health Service, 1101 Wootton Parkway, Suite 300, Rockville, MD 20852, USA

**Keywords:** hand hygiene, infection prevention, healthcare facilities, Belize

## Abstract

Access to hand hygiene (HH) resources in clinical settings is important to prevent healthcare-associated infections, including COVID-19. However, many countries, including Belize, have limited national data on the availability of HH resources and healthcare worker (HCW) hand hygiene adherence (HHA) in healthcare facilities (HCFs). We conducted a study in the 11 largest public HCFs across Belize to evaluate access to HH resources and HHA before and after an intervention (provision of alcohol-based hand rub (ABHR) wall mounts and HH training). Descriptive statistics and multilevel logistic regressions were used to assess changes in HH resources and HHA from baseline to follow-up and explore factors associated with HHA. There was a 19 percent increase in rooms with functional wall-mounted ABHR dispensers (44% to 63%) post-intervention. HHA did not improve from baseline (52%) to follow-up (50%). Combining baseline and follow-up data, HHA was higher when ABHR and soap and water were present (aOR = 4.19, 95% CI = 2.11, 8.32) and when only ABHR was present (aOR = 3.85, 95% CI = 1.92, 7.72) compared with when soap and water were present alone. The decreased perceived risk of COVID-19 at follow-up may explain the null HHA findings. However, our assessment of HH resources and practices provides a useful foundation for future HH programs in HCFs.

## 1. Introduction

Healthcare-associated infections (HAIs) account for an estimated 10 out of every 100 patient hospitalizations in low- and middle-income countries (LMICs) [1]. Hand hygiene (HH) in healthcare facilities (HCFs) is critical to reduce the risk of HAIs [2,3]. During the COVID-19 pandemic, HH was an important public health measure to mitigate the risks of infection [4,5]. It is estimated that 15–30% of HAIs can be prevented by proper HH practices in HCFs [6].

Access to HH resources is necessary but often limited in LMICs, especially during the COVID-19 pandemic [7]. Alcohol-based hand rub (ABHR) is the recommended HH method for most healthcare settings, including during times of increased patient volume or infectious disease outbreaks, such as the COVID-19 pandemic [8]. ABHR is a cost-effective solution to a gap in HH resources as it provides a quick and simple way to practice adequate HH in HCFs regardless of access to soap and water [9]. Despite evidence demonstrating the importance of HH and its impact on reducing HAIs in HCFs, studies have found low hand hygiene adherence (HHA) in LMICs, contributing to a high burden of HAIs [7,10].

Belize is an upper-middle-income country in Central America with a Human Development Index ranking of 118 out of 193 countries as of 2024; Belize has a high poverty rate (52%) and large income disparity [11,12]. Populations living in poverty have a higher risk for infectious diseases, underscoring the importance of public HCFs providing safe settings where citizens can access healthcare [13]. There is a dearth of data regarding HH in Belizean HCFs, particularly in the last decade. Berendes et al. reported baseline HH observation data from this study as part of COVID-19 emergency response efforts but did not include follow-up data that are provided here [7]. Other systematic reviews and studies that evaluated HH in HCFs in Latin America and the Caribbean reported poor HH practices in the region, but presented no data from Belize [14,15]. A two-part intervention of ABHR wall mounts with bottles and online HH training for HCWs was introduced in 11 public HCFs in Belize to improve HHA among HCWs. This study aimed to assess HHA among healthcare workers (HCWs) in public Belizean HCFs before and after the introduction of this HH intervention and identify factors associated with HHA.

## 2. Methods

### 2.1. Study Design

This study was implemented at 11 public hospitals and clinics across all six government districts of Belize participating in the existing acute febrile illness surveillance system, an ongoing collaboration between Baylor College of Medicine (BCM), the Belize Ministry of Health and Wellness, and the U.S. Centers for Disease Control and Prevention (CDC). The 11 HCFs include 4 polyclinics and 7 hospitals. Polyclinics mainly provide outpatient services with the average number of monthly outpatient consultations ranging from 865 to over 2200. The hospitals provide both outpatient and inpatient services. Monthly outpatient consultations at these hospitals range from 160 to 3400, while monthly admissions range from 160 to 2100.

At each participating site, we conducted a baseline facility assessment of available HH resources and observed HH practices among HCWs at baseline to understand access to and use of HH resources by HCWs. An intervention was developed based on the results of the baseline assessments and was provided to all participating HCFs. A follow-up assessment using the same data collection methods as the baseline assessment was performed to evaluate the impact of the intervention. This study was approved by the BCM Institutional Review Board (Protocol H-49250) and the Belize Ministry of Health and Wellness Ethics Committee. This activity was reviewed by CDC, deemed not research, and was conducted consistently with applicable federal law and CDC policy (45 CFR 46.102(l)).

### 2.2. Facility Assessment

Baseline facility assessments were conducted at the 11 participating HCFs during June and July 2021, and follow-up data collection was conducted in June 2022. The assessments were conducted in person by trained BCM enumerators. The assessment at each facility included all patient care rooms accessible at the time of the visit. A room was defined as an area separated from other areas by a wall; an area with multiple patient beds was considered one room if beds were only separated by curtains. In each patient care room, the enumerator documented general information about the room, such as the type of room, the department in which the room is located, and if any of the following activities occur in the room: physical contact between provider and patients, handling of laboratory specimens or medications, overnight patient stay, donning and doffing of personal protective equipment, or patient check-ins. Additionally, the enumerators recorded the type of handwashing station (HWS) present in the rooms, the functionality of any taps present, the availability of water at the HWS, and the availability of soap (within 3 feet of the HWS), and drying materials (within 3 feet of the HWS) at the time of the assessment. A HWS was defined as a stationary structure (e.g., a sink) or container used for handwashing. A functional HWS was considered functional if it had water and soap. The enumerator also assessed each of the ABHR dispensers in the patient care rooms. An ABHR dispenser was defined as any ready-to-use bottles or cartridges used to dispense ABHR. For each ABHR dispenser, the following items were documented: the type of ABHR dispenser, where the dispenser was stationed, the volume of the dispenser, the presence of ABHR inside the dispenser, and the functionality of the dispenser. A functional ABHR dispenser was defined as a dispenser that was mechanically operating with ABHR in the dispenser at the time of the assessment. The assessment did not include personal ABHR containers that healthcare providers had on their person. All facility assessments were recorded electronically in REDCap [16,17].

### 2.3. Hand Hygiene Observations

HCWs’ HH practices before and after patient contact were observed by trained BCM enumerators. The baseline observations were conducted one time at each facility in July 2021, and the follow-up observations were conducted one time at each facility during June–August 2022. Verbal consent was obtained from each HCW before the observations. To minimize bias in HH practices, the HCWs were informed that the observations were to document the frequency of different medical procedures at their facility. The number of HCWs needed to observe per facility was calculated based on the number of healthcare workers employed at the facility. The calculation assumed HHA increased from 50% at baseline to 65% at follow-up, with 80% power, and a 95% confidence interval. Healthcare workers were chosen for observation by convenience sampling. The HH observation tool was adapted based on the WHO’s Five Moments for Hand Hygiene [2]. For each HCW, the enumerators observed three patient contacts or up to one hour, whichever happened first, before observing the next healthcare worker. Each patient contact was defined as physical interaction between the HCW and the patient. A patient contact started at the point of first physical contact with the patient and ended when the patient left the room, the HCW left the room, or the HCW touched anything other than the patient or medical supplies. Thus, multiple patient contacts were possible for a single patient/provider consultation. For each patient contact, the enumerator documented the HCW role, the location of the observation, the HH materials present in the room, whether the procedure was invasive (e.g., contact with broken skin or mucous membranes) or non-invasive, and whether the HCW washed hands with soap and water, washed hands with water only, used ABHR, or did not wash hands or use ABHR) before and after contact with the patient. Each instance of before patient contact or after patient contact was defined as an HH opportunity. HHA was defined as either handwashing with soap and water, or using ABHR on the hands. Hand hygiene observations were recorded on printed logs and then entered into REDCap. As this was an adapted version of WHO’s Five Moments for Hand Hygiene Observation Form, certain HH opportunities, such as between body fluid exposure and aseptic procedures or between body fluid exposure and touching a patient, may have been missed.

### 2.4. Intervention

**Figure 1:**
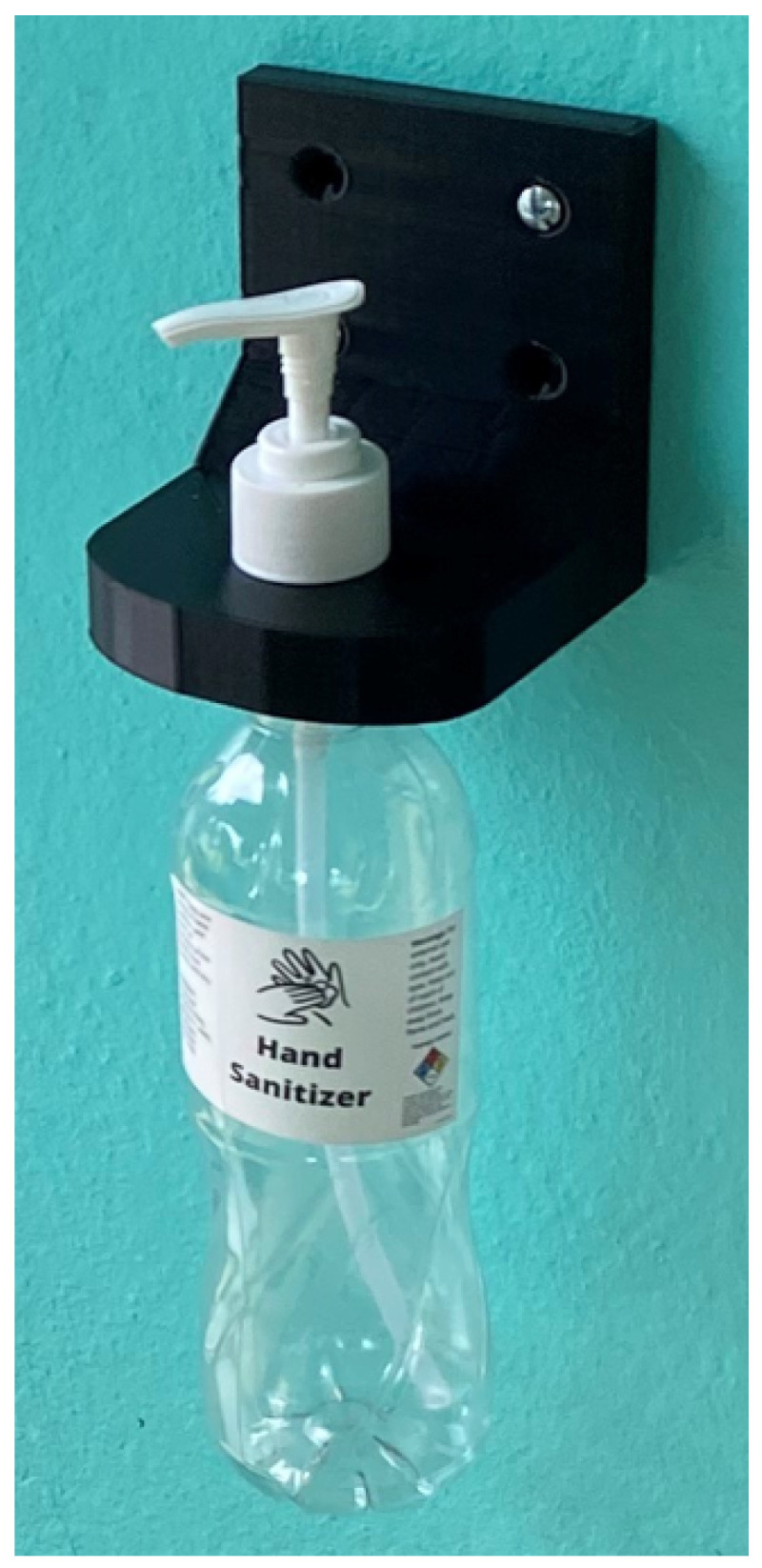
ABHR mount and labeled bottle distributed to facilities.

An intervention was developed based on the baseline data to improve access to HH materials and healthcare workers’ knowledge of HH practices. ABHR wall mounts, bottles, pumps, and sticker labels were distributed to each facility to install in rooms where ABHR was not available (Figure 1). The quantity distributed to each facility varied based on gaps identified from the facility assessments. The wall mounts were 3D printed at BCM using durable plastic and were designed to fit commonly used water bottles in Belize and easily sourced bottle pumps. The wall mounts, bottles, pumps, and bottle labels were distributed to facilities in March 2022. The wall mounts were installed at 8 of the 11 participating HCFs by the time of the follow-up assessment.

An online training tool was also developed and distributed to all staff at the participating facilities. The training content was developed based on HH standards from the World Health Organization’s Five Moments for Hand Hygiene [2]. The training was programmed in REDCap to deliver interactive content, including videos of scenarios and automatic feedback on responses. The training was in English and took approximately 20 min to complete. Healthcare workers were required to take a pre-quiz before the training module to demonstrate their existing knowledge of HH. The training then covered content such as the importance of HH in HCFs, critical moments for HH practice, proper techniques for ABHR use and handwashing, when to use ABHR instead of washing hands with soap and water and vice versa, and how to don/doff gloves properly. After the training, a post-quiz was required to document healthcare workers’ understanding of the training content; a score of 70% on the post-test was considered a passing grade. For healthcare workers with difficulty accessing the online training, an adapted paper version of the training was provided one month before follow-up evaluations, and the pre-quiz and post-quiz responses were also recorded on paper. The training was launched in January 2022 and was available through May 2022. During this period, a weekly email update was sent to administrators at each participating HCF to encourage participation.

### 2.5. Statistical Analysis

Hand hygiene resources in rooms where HH was deemed critical were compared between the baseline and follow-up assessments. The following criteria were used to identify rooms where HH was critical: (1) healthcare providers have physical contact with patients in the room, (2) staff handle lab specimens or medications in the room, (3) patients stay in the room overnight, or (4) patient check-in or triage occurs in the room. Descriptive statistics and percent changes between baseline and follow-up were calculated for rooms with a HWS with water only, a HWS with water and soap, any ABHR dispensers, and functional ABHR dispensers. The presence of a functional ABHR dispenser was also compared between baseline and follow-up by facility type, the health region where the facility was located, the type of room, and HCF. Chi-square and Fisher’s exact tests were used to assess the significance of the change from baseline to follow-up.

The HHA of healthcare workers was also compared between baseline and follow-up. Descriptive statistics were calculated for HHA during all opportunities where HH activities should be performed and where either soap and water or ABHR was available. In a single patient interaction, there are a minimum of two HH opportunities, before patient contact and after patient contact. Bivariate and multivariable analyses were performed to assess associations between HHA and facility type, number of clinical staff (as a proxy for HCF size), health region, HCW role, procedure type, moment of HH opportunity, and study timepoint using aggregate data from baseline and follow-up. Odds ratios and 95% confidence intervals were calculated using multilevel logistic regression to account for potential clustering within each health facility. The multivariable model (model 1) was constructed to include all variables that were considered to be potentially important predictors of HHA: timepoint, facility type, health region, HCW role, procedure type, and moment of HH opportunity. Another multivariable model (model 2) was constructed to evaluate the odds of HHA when different HH materials were present, adjusting for facility type, health region, HCW role, procedure type, and moment of HH opportunity. A sensitivity analysis was performed excluding HCFs where ABHR mounts were not installed during the intervention period (Table S1). All statistical tests were considered significant at the 0.05 level. All analyses were performed using Microsoft Excel (Microsoft, Redmond, WA, USA) and STATA version 16.0 (StataCorp, College Station, TX, USA).

## 3. Results

### 3.1. Facility Assessment

Across all 11 facilities, a total of 270 patient care rooms were assessed at baseline, and 331 rooms were assessed at follow-up (Table 1). Approximately two-thirds of patient care rooms had a functional HWS at baseline (*n* = 177, 66%) and follow-up (*n* = 229, 69%) (*p* = 0.339), (this was not part of the intervention). There was a 19-percent increase in the proportion of rooms with a functional ABHR dispenser, from 44% at baseline to 63% at follow-up (*p* < 0.001). The percentage of patient care rooms with either a functional HWS or a functional ABHR dispenser significantly increased from 79% at baseline to 86% at follow-up (*p* = 0.043).

The percent change in the proportion of rooms with a functional ABHR dispenser increased significantly at 5 of the 11 facilities, ranging from 19 to 57 percent (Table 2). Among polyclinics, the proportion of rooms with a functional ABHR dispenser was 37% at baseline compared with 55% at follow-up (*p* = 0.034). The proportions of hospital rooms with a functional ABHR dispenser increased from 47% at baseline to 66% at follow-up (*p* < 0.001). The percentage of rooms with a functional dispenser increased between baseline and follow-up in all health regions, with the largest increase in the Western region (36%, *p* < 0.001) and the smallest increase in the Southern region (6%, *p* = 0.532). The proportion of rooms with a functional ABHR dispenser in rooms where provider–patient contacts occurred, staff handling specimens/medications, and patients staying overnight increased by 16–22 percent.

### 3.2. Hand Hygiene Training

Participation in the online HH training varied by facility (Table 3). The median proportion of staff completing the training was 44% (range 6–120%). Due to staff turnover, some numbers under this column are greater than the total number of staff. The pass rate among training participants (scored ≥ 70% on the post-quiz) also varied, ranging from 36% at Facility 3 to 95% at Facility 7.

### 3.3. Hand Hygiene Observations

A total of 1439 HH opportunities were observed, 669 at baseline and 770 at follow-up. Overall, 1170 (81%) were in hospitals, and 966 (67%) were during non-invasive procedures (Table 4). The largest proportion of HH opportunities was observed among nurses (*n* = 758; 53%) and physicians (*n* = 439; 31%). HHA by individual HCF is shown in Figure S1. There was no clear relationship between HH training completion and HHA by HCF.

HHA did not change significantly between baseline (52%) and follow-up (50%) (aOR = 0.87, 95% CI = 0.69, 1.10) (multivariable model 1, Table 4). HHA was significantly lower among nurses (aOR = 0.52, 95% CI = 0.40, 0.68) and lab technicians (aOR = 0.31, 95% CI = 0.19, 0.50) compared with physicians. Staff were significantly more likely to practice HHA after patient contact compared with before patient contact (aOR = 2.09, 95% CI = 1.67, 2.61). Additionally, in multivariable model 2, HHA was higher when ABHR and soap/water were present (aOR = 4.19, 95% CI = 2.11, 8.32) or when only ABHR (aOR = 3.85, 95% CI = 1.92, 7.72) was present compared to when only soap and water were present. A sensitivity analysis excluding the three HCFs where the intervention ABHR mounts were not installed during the intervention period showed similar trends (Table S1).

## 4. Discussion

In HCFs that fully implemented the intervention, access to ABHR significantly increased between baseline and follow-up. Despite the implementation of an interactive online training tool for HH, we did not see significant improvement in HHA between baseline and follow-up, overall or within the subset of HCFs that fully implemented the intervention. Subsiding concern for the COVID-19 pandemic and inconsistent implementation of the complete intervention package may had an impact on the null results. When ABHR was present, HHA was significantly higher compared to when only soap and water were present.

The provision of ABHR mounts and labeled bottles substantially increased access to functional ABHR dispensers across polyclinics and hospitals. However, three facilities did not install the provided mounts or bottles in time for follow-up evaluations due to construction issues. Had these facilities been able to install the ABHR mounts with the bottles they had been provided before follow-up evaluations, the overall average increase in functional ABHR dispensers in participating HCFs may have been higher. Despite not having the intervention ABHR mounts, one facility had a significant increase in the proportion of rooms with functional ABHR mounts from baseline to follow-up. This may be due to the inclusion of a temporary flu clinic space at baseline, where there were limited hygiene resources, which was not in place at follow-up. Introducing large-scale interventions at the hospital level nationally can be difficult [18]. The increased access to ABHR as a HH resource makes HH more feasible for HCWs in times of need due to its convenience, accessibility, and ability to safely remove harmful contaminants [19,20]. In similar studies in Uganda and Sierra Leone, ABHR was the preferred HH method when both soap and ABHR were readily available [21,22]. The durability of the mounts and bottles of this intervention will extend access to ABHR beyond the study period. Additionally, using ABHR is the preferred HH method for routine hand antisepsis in most clinical situations [23].

Despite improved access to HH resources for HCWs in these facilities, and successful completion and high pass rates of the online training in some sites, observed HHA did not improve after the intervention. Other studies have recommended targeting a subset of HCWs, such as circulating nurses and anesthetists, who are more likely to have higher rates of interaction with patients and other HCWs, as a more effective approach to increase overall HHA [24]. Completion and pass results for our online training varied by facility. The proportion of HCWs who passed the post-training quiz was lower at some facilities, and many who failed did not repeat the training as intended. Completion could have been low due to the high turnover of HCWs in these facilities, which could have directly or indirectly affected HHA, and a lack of stable internet connection. A PDF version was provided one month before follow-up evaluations, but this may not have been sufficient time to complete the training. Repeating educational modules at regular intervals to improve knowledge and influence behavior, rather than these being given in a single instance as was carried out in our study, is recommended [2,3,25]. While participating in a single training session has limited effectiveness [26], previous participation in HH training has led to higher self-reported HH practices and participation in training programs has led to significant increases in observed HH [25,27]. In addition, the WHO recommends a multimodal intervention approach that consists of at least three or more, but usually five of the following elements: system change, training and education, monitoring and feedback, reminders and communications, and a culture of safety [2,3]. A study in India found a significant increase in HHA in two intensive care units at a tertiary care hospital after a multimodal intervention including tailored classes, strategically placed HH signs, and monitoring with verbal feedback [28]. Perhaps if our intervention had included more elements of the WHO-recommended multimodal approach, increased HHA could have been achieved. If only one additional element were chosen, reminders and communication could have a greater impact with little funding [29]. Loftus et al. recommend that staff repeat training on HH in healthcare settings and include it as part of a larger infection control program within the HCF [30]. This model could be adapted for these large public HCFs in Belize where infection control trainings and monitoring already exist.

A lack of improvement in HHA could be due to HH practice fatigue or decrease in perceived risk of COVID-19 by HCWs. Studies in Guatemala and the Dominican Republic reported similar decreases in observed HHA among HCFs as the COVID-19 pandemic waned [31,32,33]. Baseline HHA was higher in our study at 51% compared with 23% in the Dominican Republic [32] and 40% in Guatemala [31]; therefore, it was not unusual that HHA in Belize would decrease as the pandemic subsided. Studies have found that health messaging related to COVID-19 became uninteresting to HCWs over time [34,35]. Baseline assessments were conducted one year into the pandemic after Belize had experienced one wave of COVID-19 infections and the follow-up assessments took place more than two years after the start of the pandemic [36]. Additionally, the increase in availability of the COVID-19 vaccine may have influenced the uptake of non-pharmaceutical interventions, such as HH. By July 2021 (baseline), only 22% of the Belizean population had received at least one dose of the COVID-19 vaccine compared to 59% in June 2022 (follow-up) [37]. Furthermore, perceived risk over time may decrease, especially for HCWs in LMICs dealing with many types of infectious disease risks [35]. While the ABHR mounts and HH training were not exclusively designed for response to the COVID-19 pandemic, many HCWs may have viewed it that way given the timing and general messaging throughout the country and within HCFs.

There were no significant differences in HHA between facility types (hospitals vs. polyclinics) or health regions, indicating that neither level nor location of the HCF influenced HHA. A similar study that provided ABHR at points of care compared HH compliance between two different departments of the same HCF and found no significant difference in HH compliance [38]. However, HHA was significantly higher when ABHR was present (with and without soap and water), suggesting that the availability of ABHR increases HH practices in clinical settings. ABHR should be present in any area of the HCFs where HCWs will potentially interact with patients. Ndegwa et al. reported that HCWs found that mounted ABHR bottles were a “cue to action” and made HH more feasible when placed in patient care areas [9]. This is ideal for HCWs in HCFs with unreliable access to soap and water and experiencing higher patient volumes or staffing shortages, as may have been the case during our study period.

Interestingly, in our study, physicians had higher HHA than any other occupational group, significantly more so than nurses and lab technicians. Previous HH studies in Nigeria, Ethiopia, and comparisons of five LMICs have found that typically nurses are more likely to practice HH than physicians [39,40,41]. Systematic reviews of HH in HCFs have also consistently found higher HHA among nurses compared with physicians [42,43]. Consistent with our findings, a study in a Kenyan newborn unit also found significantly lower compliance among nurses compared with physicians and an HH study in Ugandan HCFs found doctors practiced HH more than twice as often as nurses and lab techs, along with a study comparing HCWs in five LMICs including Belize [22,44,45]. HHA was found to be significantly greater after patient contact than before contact across all HH opportunities observed. This is a common finding in the literature [7,14,42,43,44]. HCWs were significantly more likely to practice HH for invasive procedures, as has been found in other studies as well [46]. HCWs in India were observed to practice HH significantly more after invasive procedures compared to after non-invasive procedures [46]. Since HCWs are more prone to practice HH for invasive procedures and after interacting with a patient, there seems to already be an understanding of potential contamination in those scenarios [46]. Future HH education for HCWs should focus on hand contamination in non-invasive procedures and before patient contact.

In this study, we were able to include HCFs from all districts of Belize and systematically assess HH resources and practices. This is the first national assessment of its kind in Belize and provides robust data from facility assessments and HH observations. However, there are several limitations to this study worth noting. While the gold standard of directly observing HHA was used, HCWs may have inferred what the observer was documenting over time [47]. Although discretion was attempted, an enumerator’s presence alone may have biased our results, especially for the enumerators who were not Belizean and did not work in the HCFs. This bias could be minimized in the future by having a Belizean healthcare worker from a different facility shadow staff in the healthcare facility of interest for their observations. HH observations were used as an evaluation metric in this study, but for true behavior change to take place regarding HH, routine observation of HH performance, in conjunction with feedback to those observed, is recommended [2,3]. The adapted version of the HH observation tool may have also left out some of the WHO’s Five Moments for Hand Hygiene. In this analysis, we considered HHA as washing hands with soap and water or using hand sanitizer; however, future studies may consider documenting HHA based on if hands are exposed to bodily fluids. In addition, accurate measurements of training completion and pass rates were difficult to obtain due to high staff turnover in many of the HCFs and a lack of recording staff numbers at the time of training being provided. Some HCWs may have had challenges accessing the training due to limited internet in their HCF or geographic area, and the PDF version may not have reached them before follow-up evaluations. Other limitations to this study include hindrances with implementation of the intervention. Although facilities received ABHR mounts and bottles directed to place them in patient care areas, the exact quantities provided and exact placement were not directly confirmed. Additionally, the rooms selected for observing HH practices may not have been the same rooms where our ABHR mounts were placed, meaning some of the improvement in HHA may not be able to be attributed to the intervention. Due to the restructuring of some HCFs, the rooms assessed at follow-up may not have been the same rooms present at baseline. Future HH interventions could consider incorporating the remaining components of the WHO’s Multimodal Hand Hygiene Improvement Strategy to improve HHA.

## 5. Conclusions

Hand hygiene is critical to protect healthcare workers and patients from healthcare-associated infections, especially during a public health emergency. In this study, HH materials increased following a two-part HH intervention, but HHA did not improve. The higher proportion of HHA observed across time points when ABHR was present compared with when only soap and water were present suggests that increasing the availability of ABHR dispensers in patient care areas may increase HHA among HCWs. However, sustained improvement in HHA may involve integrating multiple components of the WHO’s multimodal intervention, such as providing HH performance feedback, regularly scheduled training, and HH reminders throughout the HCFs and an approach to increase impact. Hand hygiene education and systems’ change should be tailored to the local context of the healthcare facilities and existing infection prevention and control programs to increase uptake and intervention fidelity.

## Figures and Tables

**Table 1:** Hand hygiene resources at participating healthcare facilities at baseline and follow-up.

	Baseline ^1^ *n* = 270 *n* (%)	Follow-Up ^2^ *n* = 331 *n* (%)	Percent Change	*p*-Value *
Room with a handwashing station with water only ^†^	13 (5)	21 (6)	+1	0.420
Room with a functional handwashing station ^†^	177 (66)	229 (69)	+3	0.339
Room with an ABHR dispenser	158 (59)	229 (69)	+10	**0.006**
Room with a functional ABHR dispenser	120 (44)	208 (63)	+19	**<0.001**
Room with a handwashing station with water and soap ^†^ or a functional ABHR dispenser	213 (79)	283 (86)	+7	**0.043**

ABHR: alcohol-based handrub. Functional handwashing station: soap and water present. Functional ABHR dispenser: dispenser is mechanically functional with ABHR inside. Percent change: change in the percentage of rooms with the specified characteristics between baseline and follow-up. * *p*-values were calculated using the Chi-square test. ^†^ Handwashing stations with water or with water and soap were not part of the intervention. ^1^ Baseline assessments took place in June 2021. ^2^ Follow-up assessments took place in July–August 2022.

**Table 2:** Comparison of rooms with a functional ABHR dispenser at baseline and follow-up by facility type and room characteristics, Belize, 2021–2022.

	Baseline ^1^		Follow-Up ^2^		Difference	
	Total Rooms Assessed	Rooms with a Functional ABHR Dispenser, *n* (%)	Total Rooms Assessed	Rooms with a Functional ABHR Dispenser, *n* (%)	Percent Change	*p*-Value *
**Healthcare facility**						
1	21	5 (24)	26	21 (81)	+57	**<0.001**
2	17	9 (53)	52	30 (58)	+5	0.856
3 **	11	4 (36)	25	3 (12)	−24	0.167
4	19	12 (63)	22	16 (73)	+10	0.703
5	20	8 (40)	35	20 (57)	+17	0.221
6 **	22	3 (14)	15	10 (67)	+53	**<0.001**
7	64	26 (41)	26	24 (92)	+51	**<0.001**
8	46	28 (61)	46	37 (80)	+19	**0.039**
9	15	6 (40)	22	17 (77)	+37	**0.022**
10 **	22	9 (41)	29	14 (48)	+7	0.620
11	13	10 (77)	33	16 (48)	−29	0.080
**Facility type**						
Polyclinic	67	25 (37)	84	46 (55)	+18	**0.034**
Hospital	203	95 (47)	247	162 (66)	+19	**<0.001**
**Health region**						
Central	72	27 (38)	97	49 (51)	+13	0.098
Northern	38	14 (37)	78	51 (65)	+28	**0.004**
Western	110	54 (49)	72	61 (85)	+36	**<0.001**
Southern	50	25 (50)	84	47 (56)	+6	0.532
**Room type** ^†^						
Provider–patient contact	234	104 (44)	297	188 (63)	+19	**<0.001**
Staff handle specimens/medications	207	89 (43)	221	130 (59)	+16	**0.001**
Patients stay overnight	78	25 (32)	111	59 (53)	+22	**0.005**
Patients’ check-in (triage)	74	38 (51)	56	34 (61)	+10	0.220

ABHR: alcohol-based hand rub. Functional ABHR dispenser: dispenser is mechanically functional with ABHR inside. Percent change: change in the percentage of rooms with the specified characteristics between the baseline and follow-up assessments. * *p*-values were calculated using the Chi-square test or Fisher’s exact test. ** These healthcare facilities did not install the ABHR mounts by the time of the follow-up assessment. ^†^ Categories of room types are not mutually exclusive. ^1^ Baseline assessments took place in June 2021. ^2^ Follow-up assessments took place in July–August 2022.

**Table 3:** Participation of staff in the online training and percentage of staff who passed the training, Belize, 2021–2022.

Facility	Number of Staff at the Facility	Staff Who Completed Training * n (%)	Staff Who Completed Training and Scored ≥ 70% ** *n* (%)
Facility 1	147	91 (62)	77 (85)
Facility 2	316	53 (17)	32 (60)
Facility 3	39	11 (28)	4 (36)
Facility 4	45	39 (87)	21 (54)
Facility 5	499	31 (6)	23 (74)
Facility 6	40	15 (38)	12 (80)
Facility 7	184	81 (44)	77 (95)
Facility 8	152	168 (111)	144 (86)
Facility 9	30	36 (120)	29 (81)
Facility 10	179	79 (44)	57 (72)
Facility 11	109	57 (52)	48 (84)
All Facilities	1740	661 (38)	524 (79)

* The proportion of staff who completed the training among the total staff at the facility. Due to the turnover of staff, some numbers under this column are greater than the number of total staff. ** The proportion of staff who scored ≥ 70% on the training post-quiz among those who completed the training.

**Table 4:** Factors associated with HHA of healthcare workers using aggregate data from baseline and follow-up, Belize, 2021–2022.

			Bivariate		Multivariable Model 1 **		Multivariable Model 2 ***	
	Total Opportunities for HH	Handwashing with Soap or ABHR Use *n* (%)	OR (95% CI)	*p*-Value	aOR (95% CI)	*p*-Value	aOR (95% CI)	*p*-Value
**Assessment timepoint**								
Baseline	669	350 (52)	Ref	Ref	Ref	Ref		
Follow-up	770	384 (50)	0.89 (0.71, 1.10)	0.280	0.87 (0.69, 1.10)	0.249		
**Facility type**								
Hospital	1170	616 (53)	Ref	Ref	Ref	Ref	Ref	Ref
Polyclinic	269	118 (44)	0.69 (0.30, 1.58)	0.383	0.94 (0.33, 2.67)	0.910	0.88 (0.30, 2.53)	0.807
**Number of clinical staff**								
<30	209	89 (43)	Ref	Ref				
31–60	308	171 (56)	1.73 (0.60, 5.03)	0.312				
>60	922	474 (51)	1.45 (0.56, 3.75)	0.440				
**Health region**								
Central	397	153 (39)	Ref	Ref	Ref	Ref	Ref	Ref
Northern	255	145 (57)	1.61 (0.56, 4.68)	0.379	1.54 (0.41, 5.79)	0.523	1.34 (0.35, 5.17)	0.666
Western	349	200 (57)	1.84 (0.64, 5.27)	0.257	1.58 (0.42, 5.87)	0.496	1.35 (0.35, 5.13)	0.660
Southern	438	236 (54)	1.82 (0.71, 4.65)	0.209	1.54 (0.54, 4.39)	0.419	1.38 (0.48, 4.01)	0.550
**Healthcare worker role**								
Physician	439	272 (62)	Ref	Ref	Ref	Ref	Ref	Ref
Nurse	758	341 (45)	0.54 (0.42, 0.70)	<0.001	0.52 (0.40, 0.68)	**<0.001**	0.55 (0.43, 0.72)	**<0.001**
Lab technician	128	53 (41)	0.40 (0.26, 0.61)	<0.001	0.31 (0.19, 0.50)	**<0.001**	0.32 (0.20, 0.51)	**<0.001**
Patient care assistant	56	34 (61)	0.71 (0.39, 1.31)	0.277	0.79 (0.42, 1.48)	0.469	0.77 (0.41, 1.44)	0.409
Other *	58	34 (59)	0.58 (0.33, 1.04)	0.067	0.59 (0.33, 1.06)	0.077	0.60 (0.33, 1.08)	0.089
**Procedure type**								
Non-Invasive	966	477 (49)	Ref	Ref	Ref	Ref	Ref	Ref
Invasive	463	252 (54)	1.14 (0.90, 1.44)	0.280	1.40 (1.08, 1.83)	**0.012**	1.40 (1.07, 1.83)	**0.013**
**Moment of HH opportunity**								
Before patient contact	724	312 (43)	Ref	Ref	Ref	Ref	Ref	Ref
After patient contact	715	422 (59)	2.02 (1.62, 2.52)	<0.001	2.09 (1.67, 2.61)	**<0.001**	2.09 (1.67, 2.62)	**<0.001**
**Materials present**								
Only soap and water	67	12 (18)	Ref	Ref			Ref	Ref
Only ABHR	486	228 (47)	4.16 (2.11, 8.18)	<0.001			3.85 (1.92, 7.72)	**<0.001**
ABHR and soap and water	886	494 (56)	4.70 (2.41, 9.18)	<0.001			4.19 (2.11, 8.32)	**<0.001**

HH: hand hygiene, OR: odds ratio, aOR; adjusted odds ratio, CI: confidence interval, ABHR: alcohol-based hand rub. * Other: midwives, radiologists, attendants, caretakers, and pharmacists. ** Variables in multivariable model 1 included assessment timepoint, facility type, health region, healthcare worker role, procedure type, and moment of HH opportunity. *** Variables in multivariable model 2 included materials present, facility type, health region, healthcare worker role, and procedure type.

## Data Availability

All relevant data are included in this article.

## Supplementary Materials

The following supporting information can be downloaded at: https://www.mdpi.com/article/10.3390/ijerph22040514/s1, Figure S1: Percentages of hand hygiene opportunities where staff washed their hands with soap and water or used ABHR, Belize, 2021–2022; Table S1: Factors associated with hand hygiene adherence of healthcare workers using aggregate data from baseline and follow-up excluding HCFs where ABHR mounts were not installed during the intervention period (*n* = 3), Belize, 2021–2022.
