# Supplementary material for: The Influence of Improved Access to Alcohol-Based Hand Rub and Hand Hygiene Training in Healthcare Facilities on Hand Hygiene Adherence in Belize During COVID-19: June 2021–August 2022
Source: Int J Environ Res Public Health. 2025 Mar 28;22(4):514. doi: 10.3390/ijerph22040514 (PMC12026747; doi:10.3390/ijerph22040514)
Supplement: Supplementary file 1 [file ijerph-22-00514-s001.zip › ijerph-3363695-supplementary.pdf]

Supplemental Materials

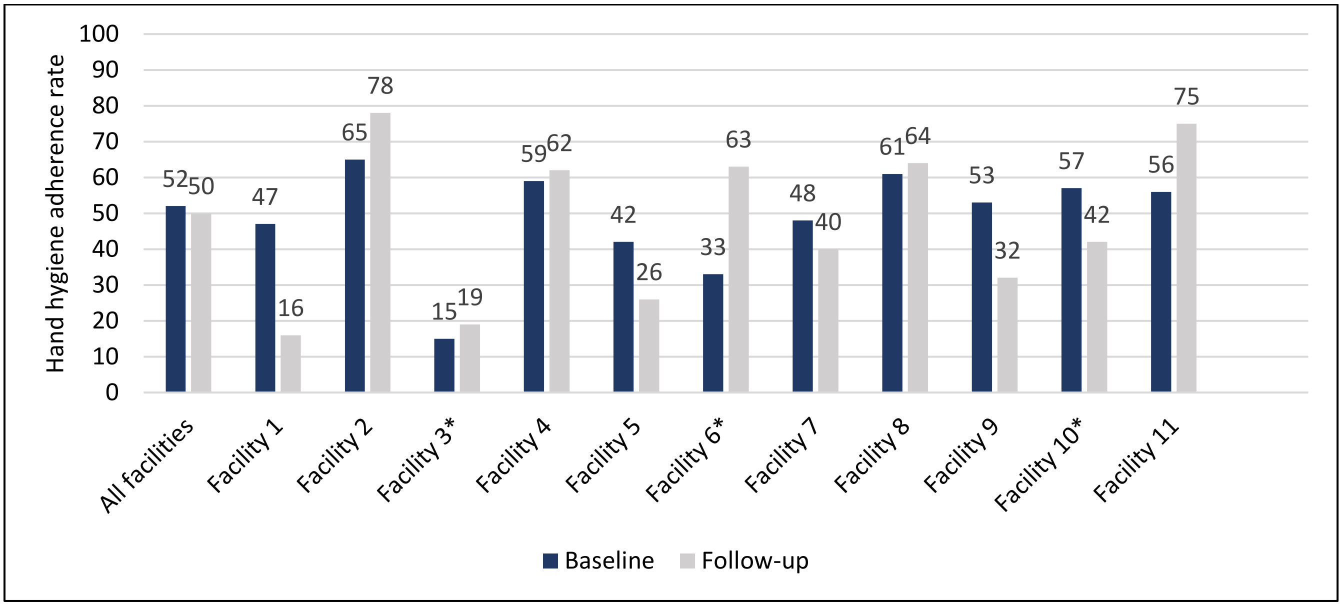

Figure S1: Percentages of hand hygiene opportunities where staff washed their hands with soap and water or used ABHR, Belize, 2021-2022

* These healthcare facilities did not install the ABHR mounts by the time of the follow-up assessment.

Table S1: Factors associated with hand hygiene adherence of healthcare workers using aggregate data from baseline and follow-up excluding HCFs where ABHR mounts were not installed during the intervention period (n=3), Belize, 2021-2022.

			Bivariate		Multivariable model 1*		Multivariable model 2**	
	Total opportunities for HH	Handwashing with soap or ABHR use n (%)	OR (95% CI)	P-Value	aOR (95% CI)	P-Value	aOR (95% CI)	P-Value
**Assessment timepoint**								
Baseline	516	281 (54)	Ref	Ref	Ref	Ref		
Follow-up	576	304 (53)	0.90 (0.70, 1.15)	0.397	0.96 (0.73, 1.26)	0.781		
**Facility type**								
Hospital	945	507 (54)	Ref	Ref	Ref	Ref	Ref	Ref
Polyclinic	147	78 (53)	1.05 (0.38, 2.91)	0.922	1.08 (0.30, 3.86)	0.903	1.08 (0.31, 3.82)	0.901
**Number of clinical staff**								
≤30	148	78 (53)	Ref	Ref				
31 – 60	248	142 (57)	1.14 (0.33, 3.85)	0.839				
≥60	697	365 (52)	0.87 (0.30, 2.52)	0.799				
**Health region**								
Central	275	113 (41)	Ref	Ref	Ref	Ref	Ref	Ref
Northern	255	145 (57)	1.20 (0.37, 3.93)	0.763	1.33 (0.32, 5.46)	0.691	1.13 (0.28, 4.59)	0.863
Western	349	200 (57)	1.37 (0.42, 4.42)	0.602	1.40 (0.35, 5.69)	0.636	1.17 (0.29, 4.70)	0.825
Southern	213	127 (60)	1.52 (0.46, 4.99)	0.489	1.46 (0.41, 5.21)	0.563	1.22 (0. 35, 4.35)	0.754
**Healthcare worker role**								
Physician	304	188 (62)	Ref	Ref	Ref	Ref	Ref	Ref
Nurse	586	294 (50)	0.71 (0.53, 0.96)	**0.027**	0.68 (0.50, 0.93)	**0.017**	0.72 (0.53, 0.98)	**0.037**
Lab technician	98	45 (46)	0.53 (0.32, 0.87)	**0.012**	0.39 (0.22, 0.67)	**0.001**	0.39 (0.23, 0.68)	**0.001**
Patient care assistant	56	34 (61)	0.83 (0.45, 1.53)	0.544	0.90 (0.47, 1.72)	0.754	0.90 (0.48, 1.72)	0.760
Other	48	24 (50)	0.42 (0.22, 0.78)	**0.007**	0.42 (0.22, 0.80)	**0.008**	0.43 (0.22, 0.83)	**0.012**
**Procedure type**								
Non-Invasive	723	369 (51)	Ref	Ref	Ref	Ref	Ref	Ref
Invasive	361	211 (58)	1.36 (1.04, 1.78)	**0.025**	1.62 (1.20, 2.21)	**0.002**	1.61 (1.19, 2.18)	**0.002**
**Moment of HH opportunity**								
Before patient contact	549	246 (45)	Ref		Ref	Ref	Ref	Ref
After patient contact	543	339 (62)	2.20 (1.70, 2.84)	**<0.001**	2.27 (1.75, 2.95)	**<0.001**	2.29 (1.76, 2.97)	**<0.001**
**Materials present**								

Only soap and water	54	11 (20)	Ref	Ref	Ref	Ref
Only ABHR	380	184 (48)	3.47 (1.67, 7.18)	**0.001**	3.48 (1.63, 7.43)	**<0.001**
ABHR and soap and water	658	390 (59)	3.97 (1.93, 8.19)	**<0.001**	3.74 (1.77, 7.91)	**<0.001**

*Variables in multivariable model 1 included: assessment timepoint, facility type, health region, healthcare worker role, procedure type, and moment of HH opportunity
**Variables in multivariable model 2 included: materials present, facility type, health region, healthcare worker role, and procedure type
